# Whole genome sequencing for copy number variant detection to improve diagnosis and management of rare diseases

**DOI:** 10.1111/dmcn.15985

**Published:** 2024-06-05

**Authors:** Pamela Bowman, Hannah Grimes, Anthony R. Dallosso, Ian Berry, Stephen Mullin, Julia Rankin, Karen J. Low

**Affiliations:** ^1^ Department of Clinical Genetics Royal Devon University NHS Foundation Trust Exeter UK; ^2^ University of Exeter Exeter UK; ^3^ Somerset NHS Foundation Trust Taunton Somerset UK; ^4^ South West Genomic Laboratory Hub North Bristol NHS Trust Bristol UK; ^5^ Department of Neurology University Hospitals Plymouth NHS Trust Plymouth UK; ^6^ Department of Clinical Genetics UHBW NHS Trust Bristol UK; ^7^ Centre for Academic Child Health, Bristol Medical School University of Bristol Bristol UK

## Abstract

First‐line genetic investigations for rare neurological and developmental conditions have limitations in their ability to detect and characterize copy number variants (CNVs). Whole genome sequencing (WGS) offers potential advantages over other methods of CNV analysis. We aimed to demonstrate the utility of CNV detection using WGS through description of three clinical cases. WGS analysis was undertaken in three patients presenting to a national rare disease service, in whom a genetic aetiology remained uncertain after gene panel testing or microarray based comparative genomic hybridization (array CGH). In all three cases, WGS identified CNVs and confirmed zygosity and pathogenicity, resulting in genetic diagnoses of *PRKN*‐related Parkinson disease, *TAOK1*‐related neurodevelopmental disorder, and *AP1G1*‐related Usmani‐Riazuddin syndrome. This case series demonstrates the value of WGS analysis in identifying or better characterizing CNVs that were missed or deemed of uncertain significance using conventional methods of testing. Importantly, our approach facilitated accurate genetic diagnosis and counselling for the families involved.

## Graphical abstract

This case series demonstrates the utility of whole genome sequencing in identifying copy number variants missed by conventional methods of testing.

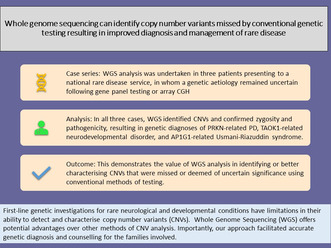

Plain language summary: https://onlinelibrary.wiley.com/doi/10.1111/dmcn.16008

**Abbreviations**
array CGH: microarray based comparative genomic hybridization
CNV: copy number variant
WGS: whole genome sequencing

What this paper adds

- Standard of care laboratory tests may fail to detect/characterize copy number variants (CNVs).
- Whole genome sequencing as a second‐line test can provide complementary information that improves detection and interpretation of CNVs.

Genomic variant detection and appropriate interpretation are crucial steps in making a genetic diagnosis, which can have significant implications for clinical management. For a variant to be clinically actionable it must be classified as likely pathogenic or pathogenic and therefore be deemed to have at least a 90% chance of accounting for the patient's phenotype.^1^, ^2^ Copy number variants (CNVs) are deletions or duplications of genomic fragments, and account for around 10% to 20% of variants causing rare disease phenotypes,^3^, ^4^ including around 7% of neurological presentations.^5^ It is therefore important to apply sensitive methods of CNV detection and interpret findings appropriately.

Conventional methods of CNV analysis, for example multiplex ligation‐dependent probe amplification, and microarray based comparative genomic hybridization (array CGH), are relatively labour intensive, expensive, and have limitations in terms of accurate detection and characterization of some CNVs.^6^ The introduction of next generation sequencing technology has enabled high throughput analysis of multiple genes simultaneously and, in some contexts, it can be an effective method for detection of CNVs.^6^ However, next generation sequencing gene panel coverage is not uniform, and targeted regions are not continuous throughout the genome, limiting the use of read depth analysis and the detection of exact breakpoints.

Whole genome sequencing (WGS) has been introduced in routine diagnostic testing in the UK for multiple rare disease indications and is accessible by mainstream clinicians including paediatricians and neurologists for the investigation of a range of adult and paediatric onset conditions. It has potential advantages over previous standard of care tests for CNV detection and interpretation for several reasons (Table 1).

**TABLE 1:** Benefits of WGS vs conventional (array CGH/gene panel/WES) methods for CNV detection.

Characteristic	WGS	Array CGH/gene panel/WES‐based CNV calling
Resolution	Genome‐wide coverage is more even. Resolution potentially down to single nucleotide using read context‐based methods. Resolution of 2–10 kb minimum is effectively achieved using read‐depth‐based methods and is agnostic to gene content/morbid status.	Minimal genome‐wide backbone/coverage outside known morbid genes at time of assay design. Coverage may be limited in regions refractory to hybridization chemistry (e.g. GC‐rich). Resolution is concentrated by assay design on coding regions of known disease genes only.
Inheritance	Trio testing is more common in WGS and will confirm phase (in recessive) and de novo aetiology up‐front, allowing better classification of variants. Parallel calling of copy number and SNP content using WGS can further refine the phase and parental origin of CNVs, potentially refining pathogenicity and recurrence risk.	Arrays typically run as a singleton assay, precluding inheritance information up‐front. Parental samples are usually requested after proband analysis, resulting in diagnostic delay. Phasing/parental origin may be possible using SNP arrays and/or from WES data but only when CNV breakpoints are serendipitously captured.
Breakpoint characterization	Breakpoints typically refined to nucleotide resolution. Precise definition of exonic content where this may be clinically critical. Ability to characterize more complex CNVs (e.g. inverted duplications, displaced duplications) by orientation, sequence content, and location of breakpoint reads.	Characterization of CNV extent is imprecise and limited to nearest probe/exon. Location and orientation of CNVs not delineated.

Abbreviations: array CGH, microarray based comparative genomic hybridization; CNV, copy number variant; SNP, single nucleotide polymorphism; WES, whole exome sequencing; WGS, whole genome sequencing.

We aimed to explore the utility of WGS as a method of characterizing CNVs in patients presenting to a national rare disease service, in whom a genetic diagnosis had not been confirmed after standard of care testing.

## METHOD

Cases were referred to the Bristol or Peninsula regional clinical genetics services for assessment and were identified for inclusion through routine clinical care. All had a neurological component to their presentation, but diagnostic uncertainty remained after initial genetic investigations. Informed consent was obtained from cases or from parents (for children) for publication of clinical data in this case series.

WGS was performed by Illumina using the TruSeq PCRfree library preparation method followed by sequencing on the NovaSeq 6000 platform (Illumina Inc, San Diego, CA, USA). Detection of CNVs of 10 kb or greater in size was performed using DRAGEN CNV (v3.2.22) (Illumina Inc). CNVs between 2 kb and 10 kb were identified using two different variant callers, DRAGEN CNV and Manta (v1.5) (Illumina Inc), where a minimum reciprocal overlap of 50% was present.

## RESULTS

### Patient 1

Patient 1 was referred to Clinical Genetics in their 40s with probable familial Parkinson disease. Clinical features and DaTSCAN imaging were consistent with Parkinson disease (Table 2). There was a family history of Parkinson disease in a maternal half‐sibling who was diagnosed in their 30s and treated with levodopa. Multiple consanguinity was reported. Next generation sequencing gene panel testing identified a copy number gain of exon 3 of the *PRKN* gene; the number of copies of the exon was calculated as between 3 and 4. Without clarification of the precise copy number, orientation, and zygosity, this CNV was classified as being of uncertain clinical significance. Of note, both duplication and triplication of exon 3 have been reported in patients with Parkinson disease;^7^, ^8^ distinguishing between a homozygous duplication and a heterozygous triplication, even in a patient with four confirmed copies of this region, is not possible with further molecular characterization. Parental samples were not available to determine phase.

**TABLE 2:** Clinical details and genetic variants identified in three cases.

Case	Age, years	Clinical features	Examination findings	Family history	Consanguinity	Medical investigations	Initial genetic investigations	WGS result
1	40–50	Presented in late 30s—pain in left arm, heaviness in right leg, slowing of movements, easy fatigue. Tightness in legs improved slightly with treatment. Type 2 diabetes. Medications: rasagaline 1 mg OD, madopar 100 mg/25 mg capsules TDS, metformin 2 g daily, atorvastatin 20 mg OD nocte	Hypomimia. Mild rigidity (more in left UL). Mild cogwheeling (left UL). No dystonic movements	Maternal half‐sibling—Parkinson disease in 30s, treated with levodopa	Yes	DaTSCAN—features suggestive of Parkinson disease or Parkinson disease plus	NGS panel test: NM_004562.2 (*PRKN*): exon 3 gain, zygosity unknown, variant of uncertain significance	NM_004562.2 (*PRKN*): exon 3 duplication, apparently homozygous, pathogenic, confirming diagnosis of *PRKN*‐associated Parkinson disease
2	5–10	Born at 37 weeks gestation via caesarean section because of breech presentation. SGA (weight 9th centile), OFC 50th–75th centiles. Recurrent ‘blue episodes’ in infancy. Global developmental delay at 12–18 months. Difficulties with social interaction. EHCP with one‐to‐one support, educational psychology input, repeated first year at school	Low muscle tone, increased deep tendon reflexes, pes planus, hypermobile joints. Mild left 2–3 syndactyly. Flattening of nose and frontal bones	No family history of relevance	No	Antenatal imaging, cranial ultrasound, echocardiogram normal	Fragile X testing, array CGH normal	Likely pathogenic heterozygous deletion at 7q11.2. 7 NM_020791.4 (*TAOK1*):c1704 + 1492 (*1_?)del Exon 16–20), likely de novo
3	5–10	Born by SVD at 38 + 5 weeks 6 months—delayed milestones and isolated episodes of head nodding (resolved spontaneously) 18 months—gross motor delay (standing but not cruising) and hand preference Behavioural difficulties: hyperactivity and a reduced sense of danger Possible infantile spasms, left hemiplegia, toe walking. Diagnosed with cerebral palsy	Right posterior plagiocephaly without evidence of craniosynostosis or perisuture ridging. Tip‐toe walking and left sided stiffness, likely secondary to scarring from old embolic infarct. Spasticity in the Achilles tendon	Sibling reported to have motor delay (walking) and assessed for autism spectrum disorder	No	Antenatal scans, sleep EEG, echocardiogram, ECG, and plasma acylcartinine levels normal. Brain MRI—scarring in the peritrigonal white matter probably representing an embolic infarct of placental origin	Array CGH—209kB deletion at 16q22.2, variant of uncertain significance	209 kb 16q22.2 interstitial deletion encompassing *AP1G1*, likely de novo. WGS showed that the deletion encompasses *AP1G1*. Likely pathogenic

Abbreviations: array CGH, microarray based comparative genomic hybridization; ECG, electrocardiogram; ECHP, education health and care plan; EEG, electroencephalogram; MRI, magnetic resonance imaging; NGS, next generation sequencing; OD, once daily; SVD, spontaneous vaginal delivery; TDS, ter die sumendum (three times daily); UL, upper limb.

Subsequent WGS testing, capturing the breakpoints and orientation of the copy number gain, was able to confirm a homozygous tandem duplication of *PRKN* exon 3, predicted to result in a truncated protein. Additionally, single nucleotide polymorphism analysis in the duplicated region and the wider locus indicated autozygosity. This allowed reclassification of the variant to likely pathogenic, confirming the genetic diagnosis of autosomal recessive *PRKN*‐related Parkinson disease. *PRKN* accounts for 1% of all adult onset and 4.6% to 10.5% of early onset Parkinson disease cases; it has a median age at onset of 31 years and a slowly progressive clinical course.^9^ The genetic diagnosis of *PRKN*‐related Parkinson disease was consistent with the clinical presentation and family history, and facilitated accurate genetic counselling for the patient in relation to prognosis and risks to wider family members, including children and siblings.

### Patient 2

Patient 2 was referred at 5 to 10 years of age with global developmental delay, hypotonia, unsteadiness, increased deep tendon reflexes, pes planus, joint hypermobility, and some mild dysmorphic features (Table 2). At birth, head circumference was large relative to weight (50–75th centile vs 9th centile). Recurrent blue episodes were documented in infancy; however no cause was identified. There was a family history of similar clinical features. Initial genetic investigations including fragile X testing and array CGH failed to identify a genetic cause. Subsequent trio WGS identified a likely pathogenic heterozygous 202.5 kb deletion on chromosome 17q11.2 encompassing seven protein‐coding genes, including *TAOK1*.

Despite the size of this deletion being within the resolution of array CGH testing, it was not visible even on reassessment of the prior array CGH data. Many array platforms have resolution deserts within regions that do not contain known disease genes, or where the array design predates the gene‐disease association, as in this case. *TAOK1* is a serine/threonine‐protein kinase required for typical neuron development via phosphorylation of tau and coordination of microtubule rearrangement.^10^ Heterozygous variants are associated with macrocephaly, hypotonia, and a variable spectrum of developmental delay and learning difficulties.^10^, ^11^, ^12^ The variant was therefore considered to explain the child's clinical features. The variant was not present in either parent therefore is likely to have arisen de novo. However, given the possibility of gonadal mosaicism, the family were offered prenatal testing for future pregnancies.

### Patient 3

**FIGURE 1:**
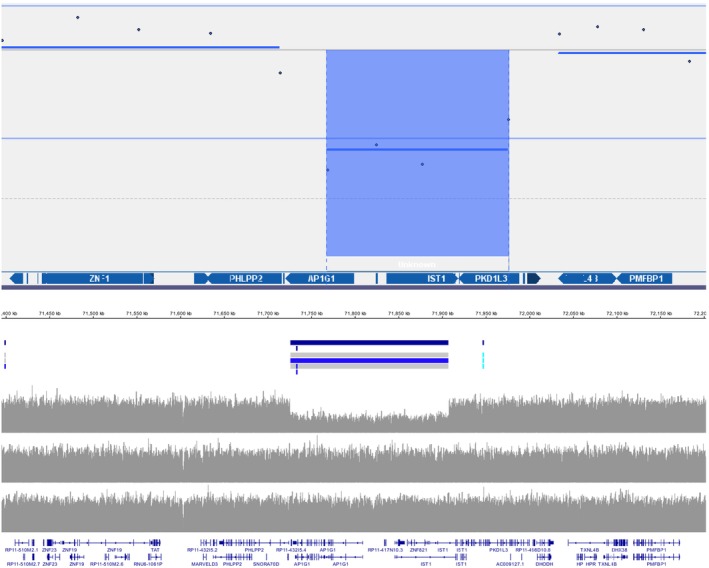
Comparison of dosage at the *AP1G1* chromosome 16 locus using different dosage analysis methods. The upper panel shows the result of singleton microarray analysis (ISCA_60kv4.2 oligonucleotide array microarray platform and Cytosure Interpret software [Oxford Gene Technology, Kidlington, UK]). Data points indicate log2 ratio dosage of individual oligonucleotide probes and the calculated minimum region of the copy number imbalance is shaded blue. Note that only a single probe is located within the *AP1G1* gene, therefore the full extent of the deletion and loss of function could not be inferred. The lower panel shows the result of whole genome sequencing dosage analysis: the location of imbalances are identified by the MANTA copy number variant (CNV) caller (horizontal blue line) which utilizes read context to precisely delineate the breakpoints, and by the sequence read depth (grey Manhattan plots for proband, mother and father at top, middle and bottom respectively), which clarify that the de novo deletion encompasses the whole of the *AP1G1* gene, allowing reclassification of the CNV as likely pathogenic.

Patient 3 was referred at 5 to 10 years of age because of developmental delay, behavioural difficulties, possible infantile spasms, left hemiplegia, and toe walking. Brain magnetic resonance imaging showed an embolic infarct thought to be placental in origin, leading to a diagnosis of cerebral palsy. Electroencephalogram was normal. Clinical features included hypotonia and developmental delay and genetic investigations were undertaken. Array CGH of the patient, and subsequently parents, identified a de novo 209 kb contiguous gene deletion variant of uncertain significance at 16q22.2. However, the extent of the CNV and involvement of the *AP1G1* gene was equivocal as only 1 out of 4 array CGH probes overlapped the gene (Figure 1, upper panel). The child was reviewed later in Clinical Genetics and was noted to have epicanthic folds, a wide mouth, and bilateral clinodactyly with parents reporting ongoing concerns regarding communication and learning. A diagnosis of attention‐deficit/hyperactivity disorder was subsequently made. Trio WGS activated at the age of 4 years upgraded the 16q22.2 variant of uncertain significance to likely pathogenic because of accurate location of breakpoints showing a whole‐gene deletion of *AP1G1* (Figure 1), and new gene‐specific information becoming available in the intervening time. Variants in the *AP1G1* gene have recently been associated with Usmani‐Riazuddin syndrome, which comprises intellectual disability, epilepsy, and developmental delay.^13^ This genetic diagnosis enabled the family to access more support for the child. The WGS analysis allowed phasing of sequence variants at the locus to demonstrate the CNV arose on the maternal allele, and showed that there was no evidence for mosaicism or a predisposing maternal balanced structural rearrangement, therefore facilitating accurate genetic counselling regarding recurrence risk. Notably only 11 families have been reported to date. This is the first reported case with whole‐gene deletion and presents with a mild but consistent phenotype.

## DISCUSSION

Our cases demonstrate that WGS can be superior to standard of care tests in the characterization and subsequent interpretation of CNVs in adults and children with rare disease. In our series, WGS analysis enabled diagnosis of *PRKN*‐related Parkinson disease, *TAOK1*‐related neurodevelopmental disorder, and Usmani‐Riazuddin syndrome, which had significant implications for the clinical care of the patients and families concerned. These diagnoses were not identified with next generation sequencing panel testing (case 1) or array CGH (cases 2 and 3), as the genetic variants were either missed or their pathogenicity remained uncertain.

Our data supports consideration of WGS as a first‐line test in patients for whom a rare monogenic disorder is suspected, as both sequence variants and CNVs can be detected using this approach. WGS has a comparable detection rate to array CGH for CNVs^14^, ^15^ and can provide important additional context, as we described here. This must be balanced against the resources available for sequencing and interpretation of WGS data on a large scale. In some cases, conventional methods of analysis may be adequate for detection of causative variants, with consideration of WGS as an additional approach if needed. However, in patients where WGS would be indicated after negative array, up‐front WGS testing may offer a reduction in overall costs and time to diagnosis. Further research, including health economics analysis, is needed to formally evaluate this.

In conclusion, we have presented the first case series of patients from a national rare disease service where WGS has been used to detect or better characterize CNVs that were missed or deemed of uncertain significance using conventional methods of testing. This has resulted in resolution of the diagnostic odyssey for the patients concerned, and provided an opportunity to offer accurate clinical advice and genetic counselling. Our results illustrate the potential utility of WGS as a first‐line test for CNV detection in in the future.

## CONFLICT OF INTEREST STATEMENT

The authors have no conflicts of interest to report.

## Data Availability

The data that supports the findings in this case series are available in the manuscript, tables and figure.
